# Time Course of Immune Response and Immunomodulation During Normal and Delayed Healing of Musculoskeletal Wounds

**DOI:** 10.3389/fimmu.2020.01056

**Published:** 2020-06-04

**Authors:** Preeti J. Muire, Lauren H. Mangum, Joseph C. Wenke

**Affiliations:** Orthopaedic Trauma Research Department, US Army Institute of Surgical Research, Fort Sam Houston, TX, United States

**Keywords:** wound healing, fracture healing, osteoimmunology, delayed fracture healing, dysregulated inflammatory response

## Abstract

Single trauma injuries or isolated fractures are often manageable and generally heal without complications. In contrast, high-energy trauma results in multi/poly-trauma injury patterns presenting imbalanced pro- and anti- inflammatory responses often leading to immune dysfunction. These injuries often exhibit delayed healing, leading to fibrosis of injury sites and delayed healing of fractures depending on the intensity of the compounding traumas. Immune dysfunction is accompanied by a temporal shift in the innate and adaptive immune cells distribution, triggered by the overwhelming release of an arsenal of inflammatory mediators such as complements, cytokines and damage associated molecular patterns (DAMPs) from necrotic cells. Recent studies have implicated this dysregulated inflammation in the poor prognosis of polytraumatic injuries, however, interventions focusing on immunomodulating inflammatory cellular composition and activation, if administered incorrectly, can result in immune suppression and unintended outcomes. Immunomodulation therapy is promising but should be conducted with consideration for the spatial and temporal distribution of the immune cells during impaired healing. This review describes the current state of knowledge in the spatiotemporal distribution patterns of immune cells at various stages during musculoskeletal wound healing, with a focus on recent advances in the field of Osteoimmunology, a study of the interface between the immune and skeletal systems, in long bone fractures. The goals of this review are to (1) discuss wound and fracture healing processes of normal and delayed healing in skeletal muscles and long bones; (2) provide a balanced perspective on temporal distributions of immune cells and skeletal cells during healing; and (3) highlight recent therapeutic interventions used to improve fracture healing. This review is intended to promote an understanding of the importance of inflammation during normal and delayed wound and fracture healing. Knowledge gained will be instrumental in developing novel immunomodulatory approaches for impaired healing.

## Introduction

**Figure 1:**
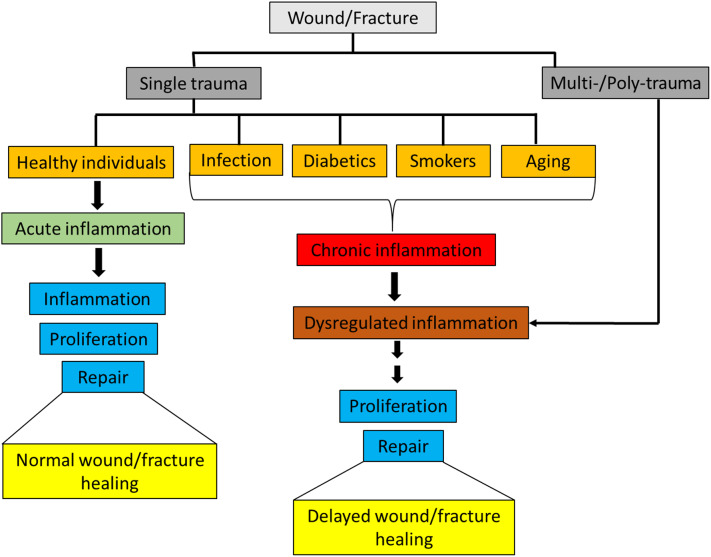
Classification of wounds/fracture and wound/fracture-healing outcomes.

Inflammation is the first response in the process of wound and fracture healing, and appropriate activation of the immune system is integral for maintaining tissue integrity and facilitating a return to homeostasis. Innate immune cells respond rapidly to injuries by both releasing and interacting with pro- and anti- inflammatory mediators [e.g., cytokines, chemokines, growth factors, and damage associated molecular patterns (DAMPs)] in order to direct the inflammatory response through inflammation and toward resolution. In healthy individuals, an isolated musculoskeletal wound or a non-critical size fracture typically heals without the need for intensive care. However, in patients with severe or polytraumatic injuries, there are major challenges associated with healing of musculoskeletal wounds, particularly in conditions where the inflammatory response is skewed or dysregulated as a result of severe injuries (1–3). Furthermore, delayed musculoskeletal wound healing also occurs in patients with pre-existing chronic inflammatory disorders caused by comorbidities such as rheumatoid arthritis, diabetes, geriatrics and smoking (4–6) (Figure 1).

Ongoing research and clinical efforts have brought about many advancements in the development of wound care products over the past decades. Several bioengineered scaffolds, primarily skin equivalents, and one cellular scaffold product, GINTUIT, have received FDA approval and have been used in the clinic to aid in wound healing and regeneration (7, 8). Furthermore, non-bioengineered scaffolds for bone regeneration, as well as the incorporation of FDA approved biologicals, like bone morphogenetic proteins (BMPs), into these scaffolds, have shown promise in improving outcomes for complicated fracture healing. In addition, investigations into the immune response to injury and the impact of immune cells on wound and fracture healing have led to a greater understanding how inflammation can influence the outcome of complicated and non-complicated injuries. These studies have helped shaped fields like osteoimmunology, and lead to the development of experimental biomaterials that can improve wound and fracture by influencing the way immune cells interact with materials.

Despite advancements in the field of regenerative medicine, there is still an urgent requirement for better wound healing therapies to improve the quality of life post-injury. However, modulation of the immune response through the use of therapeutics must be carefully considered, as failure to follow the correct therapeutic regimen could result in enhanced immune suppression and unintended outcomes. It is our belief that, in-depth knowledge of the spatial and temporal patterns of immune and inflammatory cells and their responses, post-injury, is critical for the development of effective therapeutic interventions for musculoskeletal regeneration. With this review, the authors aim to provide an in-depth survey on the innate and adaptive immune cell responses that regulate the stages of healing, with particular emphasis on the critical findings that underscore the important link between inflammation and the musculoskeletal healing in both healthy and distressed conditions. It is well known that the immune cell populations and the time at which these cells are recruited differ significantly during the healing processes of muscle and bone (9, 10). As such, and, for the ease of elucidating the spatial and temporal distribution of cells in distinct musculoskeletal tissues, we have discussed muscle wound healing and long bone fracture healing processes separately. This review seeks to address not only the known immune responses occurring within the healing cascades of these tissues under normal conditions, but also the immune responses that may be detrimental to healing in these tissues. For the purpose of this review, we have limited the scope of impaired healing in wounds and fractures to conditions of trauma, such as polytrauma and concomitant muscle loss, or to chronic, pro-inflammatory conditions associated with smoking, diabetes and aging. Finally, we highlight recent advances to therapeutic measures, including immunotherapies available to correct aberrant immune responses, that will potentially help promote timely bone regeneration. As the impact of infection on wound and fracture healing outcomes has been discussed extensively elsewhere, this topic will not be addressed in this review.

### Muscle Injury and Healing

Muscle injuries are increasingly common place and are often caused by acute trauma, with traffic accidents and armed conflict accounting for a significant portion of injuries (11). Skeletal muscle contains a pool of resident stem cells, known as satellite cells are located between the plasma membrane of myofibers and the basal lamina, which are primarily responsible for muscle regeneration (12). In addition to the expansion of satellite cells, timely and successful muscle regeneration is dependent upon a well-regulated inflammatory cascade. The complex interplay between muscle tissue and the immune system is directly responsible for the proper regeneration following soft tissue trauma. Intramuscular leukocyte populations are an essential component of healthy skeletal muscle, and these cell populations increase and change drastically following muscle injury (11, 13). Such injuries are associated with local inflammation and typically heal in the following order of events: inflammatory phase [0–7 days post-injury (dpi)]; regeneration phase (4–14 dpi); and remodeling and repair phase (14–>28 dpi).

#### Inflammatory Phase

**Figure 2:**
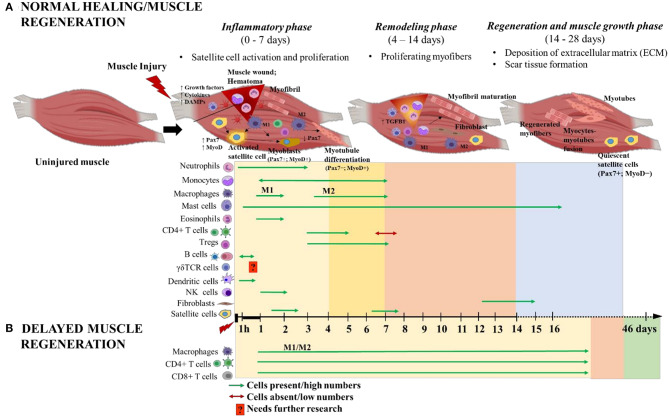
Schematic illustration of the time course of immune cells and muscle cells during **(A)** normal and **(B)** delayed muscle healing/regeneration. The three phases of normal muscle healing are inflammatory phase: 0–7 days (yellow area), remodeling phase: 4–14 days (orange area), and regeneration and muscle growth phase: 14–28 days (blue area). Overlap of inflammatory and remodeling phases: 4–7 days (bright yellow area). Paired-box transcription factor 7 (Pax7) and myoblast determination protein (MyoD) are major players during muscle regeneration and are used as markers to indicate the activated (Pax7^+^MyoD^+^), differentiated (Pax7^−^MyoD^+^) and quiescent (Pax7^+^MyoD^−^) states of satellite cells. Delayed muscle regeneration is characterized by a prolonged inflammatory phase (yellow area) with continuous infiltration of macrophages (both M1 and M2), CD4^+^ helper T cells and CD8^+^ cytotoxic T cells; a short period of remodeling-like phase (orange area) and followed by fibrosis of the muscle wound area (green area). The time scale starts at the time of injury and extends through 46 days post-injury. M1 and M2 are the two different macrophage phenotypes pro- and anti- inflammatory, respectively; and 1 h denotes 1-h post-injury. This figure was created with BioRender.com.

Following acute soft tissue injury, muscle fibers retract from the site of injury and blood from ruptured vessels enters the wound site, and forming a clot known as hematoma (11, 14, 15). The hematoma serves as a scaffold to potentiate healing and ensures that the subsequent repair occurs only within the injury site. The endogenous healing process of damaged muscle fibers involves the coordinated activities of infiltrating inflammatory cells and satellite cells responding to local and systemic cues. The inflammatory cells have a role in clearing cellular debris that are released from necrotized myofibers and they potentiate muscle regeneration. Immediately after injury, resident myeloid cells, primarily macrophages, promote the influx of neutrophils, which infiltrate within hours and peak within 12–24 h post injury (hpi) before rapidly returning to normal numbers (Figure 2). These neutrophils are responsible for initializing the inflammatory response while secreting chemoattractants that promote the infiltration of additional innate immune cells like macrophages, eosinophils and mast cells, which accumulate within the injury site at ~1 hpi−3 days post injury (dpi), indicating an acute pro-inflammatory environment (10, 16–19). Bone marrow derived monocytes traverse into the muscle injury site in response to chemoattractant signals like macrophage chemo attractant protein 1 (MCP-1), also known as CC chemokine ligand 2 (CCL2), expressed by inflammatory cells within the injured tissue (20). Monocytes are recruited by CCL7 and CCL12 to the injured tissue, where they polarize toward an M2-like wound macrophages, where they persist for 3–7 dpi and predominantly secrete insulin like growth factor1 (IGF1) to repair damaged muscle fibers (20). Some recruited monocytes are prone to differentiate into dendritic cells (DCs) in the injury site where they persist from 1 to 6 dpi and act as antigen presenting cells, bridging the innate and adaptive immune responses, by activating T cells and recruiting them to the injury site (13) (Figure 2). Moreover, the selective recruitment of increased number of CD4^+^ T helper cells, along with few CD8^+^ T cytotoxic cells, to the injury site is a hallmark of normal healing in traumatic muscle injuries (21). Starting from 3 dpi, noticeable numbers of natural killer (NK) cells and activated CD4^+^ T helper cells (enriched with T regulatory cells/Tregs [CD4^+^CD25^+^FOXP3^+^ T cells)] are selectively recruited (18) (Figure 2). In a sheep model with isolated muscle injury, B cells were observed in the muscle hematoma at 1 hpi (~6% of total CD45^+^ infiltrating lymphocytes) and they decreased at 4 hpi (~4%) and soon disappeared from the injury site (10) (Figure 2).

#### Regeneration Phase

In acute muscle injury, muscle regeneration is initiated immediately following the hematoma formation. The events occurring in the regenerative phase overlaps with most of the inflammatory phase and there is an interplay among the muscle myogenic cells and the immune cells to achieve successful muscle regeneration. The regeneration phase typically starts from 4 to 5 dpi and continues through 14 dpi. During this phase, the injury site is enriched with pro-inflammatory cytokines (TNFα and IL6) and growth factors like hepatocyte growth factor (HGF), nerve growth factor (NGF), heat shock protein 70 (Hsp70) and fibroblast growth factor (FGF) (22–24). These cytokines and growth factors (25), have a role in regulating satellite cell functions through the activation of paired-box transcription factor 7 (Pax7) and induction of myoblast determination protein (MyoD) expression (26, 27). Pax7 and MyoD are major players during muscle regeneration and are used as markers to indicate the activated (Pax7^+^MyoD^+^), differentiated (Pax7^−^MyoD^+^) and quiescent (Pax7^+^MyoD^−^) states of satellite cells and to estimate age of the muscle injury. Pax7 regulates satellite cell proliferation and prevents early myogenic differentiation and apoptosis. Pax7 expression is sharply downregulated before satellite cell differentiation (27). Whereas, MyoD is expressed early in myogenesis to initiate proliferation and differentiation of satellite cells during muscle regeneration (28). Both Pax7 and MyoD cooperate with each other during satellite cells differentiation, myoblasts formation and myoblasts fusion to generate multi-nucleated myotubes and muscle myofibers. Mutations in either Pax7 or MyoD genes significantly reduce satellite cells proliferation or inhibit satellite cells from differentiating and fusing, respectively, a condition which leads to severely impaired muscle regeneration (29, 30).

Tregs and macrophages are also integral to the muscle regeneration process. Tregs migrate toward the injury site in response to interleukin 33 (IL33) released from recruited mast cells and secrete growth factors, such as amphiregulin, required for muscle regeneration (31). Tregs are elevated from 3 to 5 dpi and expand within the injured skeletal muscles and remain there until 7 dpi (18). Additionally, Tregs interact with satellite cells via their IL10 receptor whereby they maintain the proliferation and the survival status of the activated satellite cells prior to their differentiation (18). The prolonged presence of Tregs in the injury site could possibly delay differentiation of satellite cells, suggesting the importance of time as a critical factor for the interaction between Tregs and satellite cells to drive successful muscle regeneration (18).

Macrophages can also potentiate the activation of satellite cells from a quiescent state, inducing proliferation and differentiation (11). In injured muscle, M1 pro-inflammatory macrophages phagocytose cell debris and express tumor necrosis factor α (TNFα) and IL1β. These cells peak at 2 dpi and gradually decline from 4 to 7 dpi (Figure 2). During this time, the M1 macrophages induce satellite cell proliferation, while inhibiting differentiation and fusion (32). Resolution begins 3–5 dpi and AMP-activated protein kinase 1 (AMPKα1) is required at this time for macrophage switching from M1 to M2 phenotype and to support myogenesis and muscle fiber growth (33, 34). M2 anti-inflammatory macrophages are present from 2 to 7 dpi, peaking at 4 dpi (35) (Figure 2). M2 macrophages express IL10 and transforming growth factor (TGFβ) and favor muscle repair by promoting myoblast differentiation, fusion and myotubule formation (32, 36). During the later stage of muscle regeneration, M2 macrophages acquire a resolving phenotype that is directed toward dampening of all pro-inflammatory cytokine markers while promoting fibroblast production of extracellular matrix (ECM) such as collagen, laminins, fibronectin, and proteoglycans (37). Fibroblast infiltration and differentiation typically peaks in the latter phase of muscle regeneration, at approximately 14 dpi (Figure 2). M2a and M2c macrophages release anti-inflammatory cytokine and pro-fibrotic molecules such as TGFβ that activate fibroblasts and turn on their regulatory mechanisms. Activated fibroblasts secrete ECM components, ECM remodeling factors and potentiate additional TGFβ release via autocrine stimulation. The ECM importantly aids in regulation, maintenance and repair of myofibers. Additionally, The ECM components stabilize the injury site and serve as scaffolds to promote wound closure. Any alteration to the M2 macrophage kinetics during this stage hinders normal myogenesis and leads to increased TGFβ production, altered fibroblast, elevated amounts of ECM deposition and decreased myofibers, a condition known as fibrosis [reviewed in Mann et al. (38)]. This raises caution about administering immunomodulatory therapeutic interventions during later stages of muscle regeneration phase (37). Additionally, revascularization and repair of damaged nerves occurs during this phase.

#### Remodeling and Repair Phase

Muscle remodeling and repair phase occurs concurrently with the muscle regenerative phase. The final stages of remodeling involve muscle maturation and functional repair. Mature myoblasts in injured muscle fuse with each other or with damaged myofibers resulting in the formation of myotubes, a process that is semi-dependent on the presence of MyoD in the regenerating muscle (39). During the formation of myotubes, a group of satellite cells undergo self-renewal and eventually enter a quiescent state (Pax7^+^MyoD^−^) represented as resident muscle stem cells, ready to respond to the next episode of muscle injury (40). Following fusion of myogenic cells, the newly formed myofibers increase in size and their myonuclei move to the periphery of the fibers. The connective tissue and regenerating myofibers continue to mature and orient themselves to promote proper wound closure. Finally, the new muscle tissue is the same as uninjured muscle, not only morphologically but also functionally.

### Delayed Muscle Regeneration

Delayed muscle repair mostly occurs in chronic/severe injuries as a result of volumetric muscle loss or as in muscular dystrophy. In conditions of severe muscle injuries, chronic local inflammation and elevated numbers of activated fibroblasts persists, while the regenerative capacity of satellite cells is severely attenuated. Instead, there is elevated deposition of ECM components at the injury site, which inhibits myofiber formation and leads to replacement of muscle with fibrotic/scar tissue, a hallmark of fibrosis (38). A study in Lewis rats with severe muscle injury reported greater than normal infiltration rate of mixed M1/M2 macrophages, CD4^+^ and CD8^+^ T cells at the injury site that peaked at 3 dpi and gradually declined through 28 dpi, thereby impairing muscle regeneration (41). The study further suggested that the long-term presence of CD8^+^ T cells in muscle injury is abnormal and could be a potential therapeutic target for the resolution of inflammation in delayed healing. Further, perturbation of any of the muscle repair stages via therapeutic interventions, without detailed understanding of the time course of underlying molecular and cellular events of muscle regeneration, could lead to severely impaired muscle repair (42). For example, ablation of Hsp70 activity, during the inflammatory phase, impairs neutrophil and macrophage infiltration to the injury site. However, this activity, during the regenerative phase, causes prolonged pro-inflammation (>16 dpi), necrosis, enhanced calcium deposition and impaired muscle regeneration that lasts for several weeks post-injury (24). Taken together, these advances in our understanding about acute and chronic muscle injuries, the inflammatory responses and muscle regeneration could have implications for the development of novel therapeutic strategies for timely muscle repair and functional restoration.

## Fracture Healing

Bone healing is a complex process of overlapping stages of healing that demands functional immune-osteogenic cellular responses, which are orchestrated by several biological factors. There are four recognized phases of fracture healing: inflammatory phase—beginning with the development of the hematoma and inflammation; repair phases—formation of a soft callus; and development of a hard callus; and finally remodeling phase. The utilization of growth factors, scaffolds and mesenchymal stem cells (MSCs) as a standard biological approach for bone regeneration was traditionally discussed as the triangle concept (43). Giannoudis et al. modified the triangle concept and called it the diamond concept because of the addition of a fourth element of care, i.e., mechanical stability (43). The diamond concept includes factors like (1) osteoinductive mediators such as cytokines and growth factors; (2) osteoconductive matrix that includes scaffolds or necrotic bone within the fracture site; (3) osteogenic cells that comprise osteoprogenitor cells from periosteum, MSCs from bone marrow and endothelial progenitor cells; and (4) mechanical stability of the fracture environment to induce successful healing (43, 44). Later, Loi et al. modified the diamond concept and included the importance of inflammation as the fifth factor for fracture healing (45). Although osteoimmunology is an intriguing topic and is considered an important concept in bone regeneration in fractures, its importance was overlooked in the past, and has recently gained much attention (46). Much of the research focus has been placed on the regulation of the events in fracture healing by paracrine signaling, rather than direct cellular activities, mainly due to lack of information on the temporal distribution of innate and adaptive immune cells during fracture, and this review aims to address the issue in both normal and delayed fracture healing.

**Figure 3:**
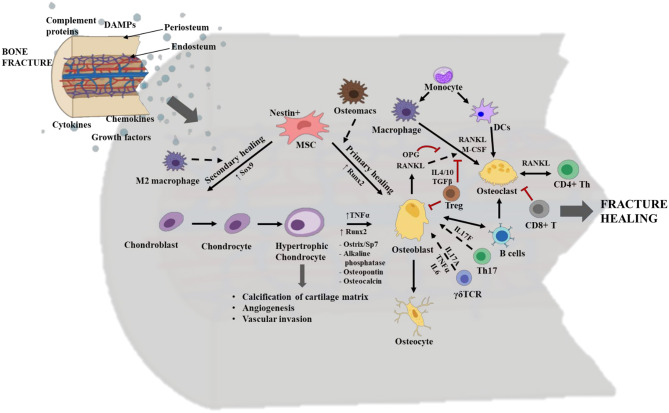
Overview of the cellular and molecular events occurring during fracture healing. The interplay between stem cells, immune cells and skeletal cells (chondrocytes, osteoblasts, and osteoclasts) is required for efficient fracture healing. In long bone fractures repair occurs via two routes: primary healing mediated by intramembranous ossification and no callus/cartilage formation or via secondary healing mediated by endochondral ossification and with callus/cartilage formation. Typically, in primary healing the mesenchymal stem cells (MSCs) directly differentiate to osteoblasts, a process regulated by the transcription factor Runt-related transcription factor 2 (Runx2) and bone marrow resident macrophages/osteomacs. Whereas, in secondary healing MSCs differentiate into chondroblast and is regulated by the transcription factor SRY-related high mobility group-box gene 9 (Sox9) and M2 macrophages. Chondroblasts further differentiate to chondrocytes, which in turn differentiate to hypertrophic chondrocytes. Hypertrophic chondrocytes have a role in calcification of the cartilage matrix, angiogenesis, and vascular invasion. They differentiate into osteoblasts, via the induction of transcription factors like Runx2 and Sp7; TNFα; and other osteogenic mediators. Osteoblasts are bone forming cells and they mature into osteocytes which mineralize the bone matrix. During repair, Th17 cells and γδ T cells promote osteoblastogenesis via the secretion of cytokines like IL17F, and IL17A, TNFα and IL-6, respectively. Osteoblasts also regulate osteoclastogenesis via production of receptor activator of nuclear factor kappa-B (RANK) ligand/RANKL and osteoprotegerin (OPG). Osteoclasts are bone resorptive cells. They belong to hematopoietic stem cell (HSC) origin and are derived from macrophages and dendritic cells (DCs) in the presence of macrophage colony stimulating factor (M-CSF) and RANKL. CD4^+^ T helper (Th) cells also secrete RANKL and crosstalk with osteoclasts in order to regulate osteoclastic activity and vice versa. Functions of osteoclasts are highly regulated by the binding of their surface receptor RANK to either RANKL for activation or OPG for suppression. Regulatory T cells (Tregs) inhibit osteoblastogenesis and osteoclastogenesis, via secretion of IL4, IL10 and transforming growth factor β (TGFβ). Activated B cells activate osteoblastogenesis and osteoclastogenesis, while CD8^+^ T cells suppress osteoclastogenesis. The dotted arrows indicate indirect role; the solid arrows indicate direct role; and the double head arrow indicates cell to cell crosstalk. The red “T” lines indicate inhibition. This figure was created with BioRender.com.

**Figure 4:**
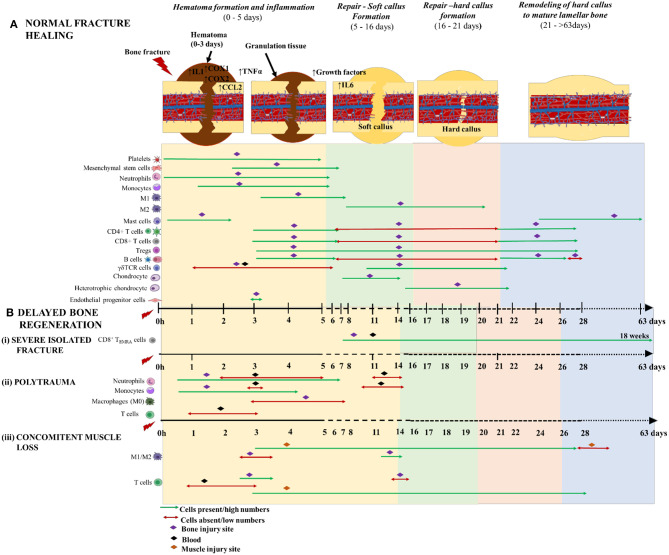
Schematic illustration of the time course of inflammatory cells, immune cells and skeletal cells during **(A)** normal fracture healing and **(B)** delayed bone regeneration in conditions like (i) severe isolated fracture; (ii) polytrauma; and (iii) concomitant muscle loss. The three phases of normal fracture healing are Hematoma formation and inflammatory phase: 0–5 days (yellow area); Repair—Soft callus formation: 5–16 days (green area); and Hard callus formation: 6–21days (orange area); and Remodeling of hard callus to mature lamellar bone: 21–>63 days (blue area). Delayed bone healing is characterized by prolonged and dysregulated inflammatory phase which causes a significant delay to repair and remodeling of bone. The time scale starts at the time of injury and extends beyond 63 days post-injury. M1 and M2 are the two different macrophage phenotypes pro- and anti- inflammatory, respectively; 1 h denotes 1-h post-injury; and CD8^+^T_EMRA_ cells (CD8^+^ terminally differentiated effector memory T cells.

There are two routes by which bone fractures can heal, depending on the fracture fixation stability. These are primary healing by intramembranous ossification, which lacks callus/cartilage formation, or secondary healing by endochondral ossification that includes chondrogenesis and the formation of fracture callus (Figure 4). Endochondral ossification, a topic that will be discussed at length in this review, is a more common mechanism of healing and is prominent in weight bearing bones with moderate stability at the fractured zone due to sheer pressure. Fracture healing is initiated by a local acute inflammatory response that includes the formation of a fracture hematoma and clearing of necrotic cell debris and DAMPs from the fracture site. This activity promotes the recruitment of MSCs and additional immune cells to the fracture site to initiate the repair phases, including the initial formation of a cartilaginous soft callus which is soon converted into a hard-bony trabecular callus or woven bone. Osteoblasts and osteoclasts are found within the soft callus, and these two cell types are pivotal in regulating bone formation and resorption, respectively. The final phase in fracture healing is the remodeling phase. Osteoblasts are bone forming cells that originate from the differentiation of MSCs during primary healing conditions, or from the trans differentiation of heterotrophic chondrocytes in secondary healing conditions (Figure 2). The differentiation of osteoblasts from heterotrophic chondrocytes and their activation status is largely influenced by the local immune cells and molecular mediators such as Runx2, TNFα, ostrix, alkaline phosphatase, osteopontin and osteocalcin (47, 48) (Figure 3). Regardless of their lineage, following the onset of differentiation, osteoblasts undergo extensive morphological changes to form cells with dendrite like extensions, called osteocytes, that make up most of the mineralized bone matrix. Osteoclasts are large, multinucleated, myeloid derived specialized cells that are necessary for breaking down bone matrix; a vital step in the processes of fracture repair and bone remodeling (49). These cells differentiate largely from mononuclear cells through the process of osteoclastogenesis. It is a complex and highly regulated process that requires RANK (Receptor activator of nuclear factor kappa-B) and its ligands, RANKL and osteoprotegerin (OPG), as well as cell-cell crosstalk with activated osteoblasts and CD4^+^ T helper cells (49, 50) (Figure 3). Importantly, early signals that influence the commitment and differentiation of MSCs to osteogenic or chondrogenic lineage as well as signals that regulate cell proliferation and terminal differentiation during later stages are critical for successful healing outcomes. Herein, we discuss the secondary healing phases of fracture healing and have attempted to cover most of the immune cells that have been investigated in various long bone fracture model studies.

### Hematoma Formation and Inflammation

Immediately following musculoskeletal injury, large amounts of DAMPs are expelled from the necrotic tissue and are present in the surrounding microenvironment of the fracture. DAMPs activate highly evolutionary conserved intracellular signaling cascades that mediates the rapid infiltration of inflammatory cells to the injury site via passive or chemotactic mechanisms from ruptured vasculature and the exposed bone marrow. The initial cellular component of the fracture environment includes platelets, RBCs, neutrophils, macrophages, T cells, B cells, regulatory B cells (Bregs), mast cells, histocytes and eosinophils to form a tight clump of cells known as fracture hematoma (51). The hematoma contributes to initiation and optimization of the healing process and throughout all stages until resolution of bone-union. It also provides structural framework and serves as a natural scaffold for osteogenesis to occur, thus suggesting the importance of retaining the hematoma at the fracture site throughout the healing process (52). Disruption of the vascular network within the facture site causes a shift in the hematoma's internal microenvironment that includes, anaerobic energy turnover causing an acidic pH environment, high sodium and potassium levels and severe hypoxic conditions, thereby depriving the surrounding environment from essential nutrient supply for proper maintenance. Despite such harsh physiological conditions, some macrophages and T cells can adapt and thrive in the hematoma by altering their phenotypes (53). The hematoma matures within 24 h post fracture (hpf) and the pH transitions toward neutral to less alkaline nature, allowing the activity of alkaline phosphatase. This change in pH affects the cell populations that make up the granulation tissues, forcing them to undergo apoptosis and causing an oxidative burst, which is detrimental to other cells, especially the progenitor stem cells. Change in pH is also responsible for the later recruitment [i.e., 2–days post fracture (dpf)] of circulating osteoblasts and periosteum derived MSCs via stromal cell derived factor 1 (SDF-1)/CXCR4 signaling to the fracture site (54, 55). Additionally, complementary pathway proteins, cytokines, chemokines, clotting proteins and growth factors have added roles in maintenance of hematoma, which maintains the integrity of the fracture healing process. The hematoma sets up the stage for the progenitors and other inflammatory cells to mediate their responses (52, 56, 57). Overall, the hematoma is integral for proper healing, and disturbing or surgically removing it will change the fracture microenvironment and delay callus formation and, consequently, bone regeneration.

Neutrophils are recruited to the fracture site from ~1 hpf through 5 dpf via sensing a chemical gradient released by chemo-attracting/pro-inflammatory mediators like growth factors, cytokines and chemokines (58) (Figure 4A). The neutrophils potentiate the inflammatory cascade of events by further secreting chemo-attractants and recruit monocytes to the hematoma. At this stage, tissue macrophages become metabolically active and switch from resting states to active macrophages (59). Monocytes and activated macrophages are prominent in the hematoma at 1–5 dpf and 3–7 dpf, respectively (52) (Figure 4A). Neutrophils and macrophages phagocytose cellular debris and DAMPs from injured tissue. Once activated, the macrophages also release chemoattractants like IL1β and TNFα to stimulate fibroblast proliferation and recruit other myeloid and lymphoid cells to the injury site (60). These cells and other granulocytes together make up the fibronectin matrix, which acts as a temporary framework in the hematoma during the healing process. The mature monocytes/macrophages and DCs within this framework eventually differentiate to osteoclasts which are pivotal in bone resorption during the fracture repair phase (61, 62) (Figure 3).

Mast cells are granulated cells found in circulation in their immature states. Apart from their roles in allergic responses and autoimmunity, they also have a positive role in regulating tissue remodeling, wound healing and fracture healing (63). Mast cells are effectors of early inflammatory signals and they elicit their regulatory functions on both innate and adaptive immune responses during all phases of fracture healing. These cells have a high affinity toward substance P and calcitonin gene related peptides, which are released from sensory neurons following injury. Large numbers of mast cells infiltrate into the injury site from 1 to 48 hpf, where they localize and degranulate in the area around the lumen side of blood vessels and in the bone marrow cavity of the endosteal callus (64, 65) (Figure 4A). Additionally, they promote vascular permeability (66) and angiogenesis (67) as well as regulate bone metabolism. Kroner et al., reported that a mouse model with depleted mast cells (Mcpt-5 Cre R-DTA mice), had decreased neutrophil invasion at 1 dpf, as well as decreased osteoclast numbers at the injury site, a finding that was associated with significantly delayed fracture healing (68). During the inflammatory phase, mast cells are required for early recruitment of neutrophils to the fracture site, maintaining osteoclast activity and regulating pro-inflammatory cytokine production (69). Upon degranulation, the mast cells release pro-inflammatory mediators such as cytokines, chymase, proteases and tryptase that contribute to allergic inflammation, but some mediators, such as histamine and VEGF, may act as mediators of tissue repair by inducing vascular hyperpermeability within the injury site (69). This explains the connection between mast cells and recruitment of early neutrophils to the injury site. In another study using a different mouse model for mast cell deficiency (Cpa3Cre/+ mice), the same authors report abnormal bone regeneration, impaired vascularization and dysregulated osteoclastogenesis when compared to wild type mice with normal mast cell production (70). This indicates that mast cells have a role in promoting bone healing by possibly recruiting vascular endothelial cells during the initial healing phases and by coordinating both anabolic and catabolic activity during bone remodeling process [reviewed in Ragipoglu et al. (71)]. However, additional studies indicate that this beneficial effect may be limited to long bones, as depletion of mast cells enhanced healing in a cranial defect model (72). Taken together, these studies illustrate that mast cells have complex, but possibly a beneficial role in fracture healing.

Information on the role of NK cells in fracture healing has not been investigated at length, although one *in-vitro* study reported suppression of NK cells when incubated with fluids from early fractured zone and plasma from human subjects (73). This observation suggests that NK cells could be absent during early hours of healing. The observation that T and B lymphocytes are recruited in a two wave fashion to the fracture site and their increased distribution throughout the systemic bone marrow suggests they are important players throughout the bone healing process (74). Typically, T cells (CD4^+^, CD8^+^ and Tregs) infiltrate the fracture site from 3 to 28 dpf (75), while B cells are reported to pour-in the fracture site from 3 to 5 dpf just at the initiation of soft callus formation (74) (Figure 4A). During this phase the T cells are activated by antigen presenting cells (DCs and macrophages) and they in turn activate B cells to initiate an adaptive immune response (76). B cells have a role in antibody production and they also function as antigen presenting cells (77). They have additional immune functions such as modulation of the cytokine production and maintenance of immunological tolerance (78). Regulatory B cells (Bregs), a subset of B cells, also have a role in promoting endogenous bone regeneration process, especially during the initial healing phases (79, 80). Bregs maintain immunological tolerance by promoting Treg survival, T helper 1 (Th1) cell suppression and inhibition of pro-inflammatory cytokines such as interferon gamma (IFNγ), TNF-α and TGF-β to promote bone regeneration (79, 81). The well-known mechanism of action of Bregs is the production of IL10, a potent anti-inflammatory cytokine (82). The functional status of Bregs is attributed to the levels of IL10 they secrete in response to inflammation and the impact of injury. In conditions of acute inflammation and normal fracture healing, the number of B regs and their IL10 levels are reportedly upregulated in circulation. Whereas, in conditions of delayed fracture healing, the number of Bregs were reduced and their IL10 levels were significantly downregulated, indicating the presence of dysfunctional Bregs (79). Further, early circulating IgM^+^CD27^+^ B cells from normal healing patients demonstrated robust suppressive function, while the later circulating IgM^+^CD27^+^ B cells did not present regulatory functions in both normal and delayed healing patients (79). Taken together, these findings indicate that dysfunctional Breg have a role in instigating delayed fracture healing and this dysfunction is accompanied by increased pro-inflammation and dysregulated lymphocyte responses. As Bregs are found in detectable levels only during acute inflammatory conditions, it is challenging to study this population under healthy conditions.

Additionally, the γδ T cells, also known as inflammatory lymphocytes have an integral role in bone regeneration [reviewed by Kalyan (83)]. In response to acute inflammatory stress signals, γδ T cells secret growth factors, promote cytokine production (IFNγ, TNFα, and IL17), induce local inflammation by recruiting macrophages and accelerate repair (84). In trauma induced severe systemic inflammatory response syndrome (SIRS), the functions of γδ T cells is reversed and the cells are severely diminished locally and in circulation especially during the initial stages of healing and are found homing in the lymphoid tissues at 3 dpi (85, 86). Immediately after injury the γδ T cells are severely diminished in the fracture hematoma and reappear in the fracture site around 10 dpf (87) (Figure 4A). This change in γδ T cell dynamics could be due to the heavy oxidative burst that is prevalent at the injury site.

### Repair–Soft Callus Formation

This stage of fracture healing is characterized by the formation of mineralized soft tissue cartilaginous matrix/soft callus formation from 7 dpf onwards (74). The soft callus is enriched with chondrocytes, collagen and proteoglycans. Other cells such as MSCs, endothelial cells, osteoblasts, osteocytes, inflammatory macrophages, bone marrow resident macrophages (osteomacs), mast cells, αβ T cells, γδ T cells, B cells and NK cells are also present in the soft callus.

Both osteomacs and inflammatory macrophages can influence bone growth and regeneration (88). Osteomacs such as erythroid island macrophages (F4/80^+^VCAM^+^CD169^+^ER-HR3^+^Ly-6G^+^), maintain immune surveillance, bone formation, functional hematopoietic stem cells niches, and erythropoiesis (89). They are also critical for differentiation of MSCs into functional osteoblasts, whereas inflammatory macrophages have an osteoclastogenic role and are critical for initiating and propagating endochondral bone formation (90) (Figure 3). Under standard healing conditions, macrophages differentiate into osteoclasts in a stable microenvironment provided by bone marrow derived stromal cells (61). Further, the undifferentiated macrophages reside in chondrogenic centers and persist there until the callus has formed and then migrate away from the injury site. The timing for macrophage elimination from the callus is very crucial to maintain proper bone regeneration. The early fracture microenvironment is usually enriched with various pro- and anti-inflammatory cytokines that might contribute to the recruitment and activation of DCs at the fracture site (91); however, this has not been investigated in long bone fractures. DCs function as professional antigen presenting cells and several studies in rheumatoid arthritis models have elucidated the importance of DC to promote bone remodeling. Similar to macrophages and lymphocytes, the immature DCs are capable of transdifferentiating into functional osteoclasts in the presence of M-CSF and RANKL (92) (Figure 3), and these osteoclasts induced bone reabsorption after *in vivo* transfer in mice (93).

T cells play a profound role in mediating osteoblastogenesis and osteoclastogenesis. At the early anabolic stage, the T cells secrete RANKL to activate osteoclasts to remove fibrin thrombus and sets the stage for chondrogenesis and later osteoblastic activity. Osteoblasts and B cells secret OPG, a decoy protein that binds to the RANK receptor on osteoclasts and suppresses their activity (94). In healthy, non-fracture conditions, CD8^+^ T cells appear in larger numbers in the bone marrow compared to CD4^+^ T cells. Conversely, in bone fractures that are healed without complications, the CD4^+^ T cell population predominates over CD8^+^ T cells, suggesting CD4^+^ T cells have a positive regulatory role in osteogenesis. Further, this indicates that the fracture significantly affects the overall T cell homeostasis in the bone and there is an inversion of relative composition of T cells during healing (74). CD4^+^ T cells are classically characterized into T helper 1 (Th1) (inflammatory) and T helper 2 (Th2) (immune modulatory) type cell responses. Additional CD4^+^ T cell subsets, such as Th17 cells and Tregs, also have immunomodulatory roles during fracture healing. Th17 cells via the secretion of IL17F promote the maturation and maintenance of osteoblasts (95) (Figure 3). While, Tregs exert an inhibitory effect on both osteoblasts and osteoclasts via secretion of IL4, IL10, and TGFβ, an important regulatory mechanism (95, 96) (Figure 3). Typically, T regs (CD4^+^CD25^+^Foxp3^+^) are pivotal in maintaining immune homeostasis (97). They suppress the immune response via the production of immunosuppressive cytokines such as IL10, TGFβ and via cell–cell contact (96, 98).

The γδ T cells can non-specifically recognize antigens from stressed cells via their innate ability. Even though γδ T cells make up ~2% of the total CD3^+^ lymphocyte population, they play a key role in the immune system's influence on skeletal injury (83). These cells can dictate Th1 and Th2 responses while they also predominantly secrete Th1 cytokines (99). Depending on their microenvironment, γδ T cells also produce Th2 cytokines and IL17 through induction of Th17 cells via the retinoic acid–related orphan receptor on γδ T cells (100). The γδ T cells are mostly activated by IL1β and IL23 and they secrete IL17A, TNFα, and IL-6 to promote osteoblastogenesis within the soft callus matrix (Figure 3). The lack of effector γδ T cell cytokines in δTCR^−/−^ KO mice is associated with impaired osteoblastogenesis, matrix production and soft callus formation (87). Further, Colburn et al. demonstrated that γδ TCR^−/−^ KO mice with a mid-tibia fracture had superior healing outcomes at the initial healing phase (i.e., 5 dpf), albeit they showed that once the other co-inflammatory mediators and cells entered the fracture microenvironment, the healing was slowed down and was not different from the WT mice. In these γδ T cell deficient mice, the early alteration on healing noted in bone and cartilage formation correlates with the reorganization of the immunologic milieu at the inflammatory stage. Therefore, at 5 dpf, the absence of γδ T cells exhibited a qualitative influence on the final bone regeneration outcome, a phenotype that did not last through 28 dpf (87). Another study reported that IL17 producing γδ T cells enhance bone regeneration following fracture (101). We anticipate that γδ T cells may have both pro- and anti-inflammatory roles and they do this by undergoing apoptosis during the initial inflammatory phase and later increase their proliferation rate as the healing process progresses into the remodeling phase, further justifying their beneficial role during the later stages of healing. Further investigation on the role of γδ T cells in fracture and their interaction with bone cells would be beneficial.

Although T cells are integral in fracture healing, interestingly the dominant lymphocyte population within the callus are B cells. They play an important role in bone metabolism and protection (74).

### Repair–Hard Callus Formation

Following the soft callus stage, the overall success of fracture healing lies in the net result of concomitant bone synthesis by osteoblasts and resorption by osteoclasts. Immune cells contribute to osteoclastogenesis and toward the appropriate bone resorption during fracture healing, thereby requiring a time dependent regulation between two very important inter-dependent steps. Osteomacs are prominent in the maturing hard callus and both inflammatory and resident macrophages potentiate anabolic processes during endochondral callus formation (88). Osteomacs are integral to the later phases of repair because they are required for osteoblast maturation and bone formation (102). Mast cells are prominent in the periosteal callus near newly formed bony trabeculae and osteoclastic bone resorption sites (68). The transition from soft to hard callus stage is also characterized by the disassembly of T and B lymphocytes from the cartilaginous areas and mostly confined to the endosteal tissue in the vicinity of the fracture gap (74). During this phase, beginning at ~14 dpf, the avascular cartilage is vascularized, and new woven bone emerges between the fracture gaps, characterized as hard callus, thus creating a bridge. Chondrocytes mature and proliferate further to become hypertrophic and begin synthesizing mineralized matrix. Between 14 and 21 dpf, large number of osteoclasts secrete the bone resorbing enzyme cathepsin K, which adheres to the newly formed bone as well as the cortices. During this phase of healing, the two subtypes of osteoblasts with different morphologies are, (i) round precursor cells diffused with the callus stroma; and (ii) palisade shaped cells lining the bone typical of an osteoblast. OPG expression is detected in the cytoplasm and in the extracellular milieu of osteoblasts (CD3^−^B220^−^) at this time (74). Further, B cells make tight cell-cell contacts with the osteoblastic cells throughout the entire callus, suggesting that, at the initiation of hard callus formation, B cells can affect the activity of both immature and fully differentiated osteoblasts (74).

Concomitantly, at 14 dpf, T and B cells reappear in large numbers in a second wave fashion via the inner vessels in the inner callus, occupying the areas nearby woven bone, but remaining still undetectable within the persisting cartilage (74) (Figure 4A). Lymphocytes in the areas of the newly formed woven bone have been shown to increase 3-fold between 14 (woven bone formation phase) and 21 (remodeling phase) dpf, with B cells outnumbering T cells during this phase of healing. Konnecke et al. (74) have demonstrated by histological analysis that the cross section of fractured bones revealed an irregular distribution of T cells throughout the length of the bone. However, within the callus area that is occupied by woven bone, the T and B cells were attached to the luminal side of the vascular endothelium, confirming their transmigration into the callus via the sinusoidal venules (74). At 14 dpf, when lymphocytes infiltrate the callus, majority of the B cells are naïve (B220^+^IgM^+^IgD^+^), and only a few memory or immature B cells (B220^+^IgM^+^IgD^+^) are detected (74). These naïve B cells appear throughout the callus and spread into the chondrocyte regions at 21 dpf. These observations imply that T and B cells are detected as early as the soft callus formation begins (i.e., 5–7 dpf), where they secrete OPG in an incremental fashion as the callus formation progresses from 14 to 21 dpf and until the remodeling phase. This suggests that the T and B cells contribute to bone formation by regulating osteoclastogenesis during the later stage of healing. Activated B cells have a role in promoting osteoclastogenesis, while CD8^+^ T cells suppressed it (103). The expression ratio of OPG to RANKL progressively increases during this phase and is significantly high at 14 dpf and peaks at 21 dpf when compared to 7 dpf, indicating the occurrence of osteoclastogenesis from 7 to 21 dpf, i.e., during maturation of the fracture callus (74). These systematic observations of the bone healing process highlight the importance of OPG and RANKL as molecular markers to track the progress of bone healing. Therefore, close monitoring of OPG and RANKL expression levels could suggest the correlation between inhibition of osteoclastogenesis and the time course of T and B cells infiltration in the callus. This information could prove helpful in terms of planning therapeutic interventions for fracture healing.

### Remodeling of Hard Callus to Mature Lamellar Bone

Bridging and stabilization of the fracture is near completion after 21 dpf, as seen in preclinical fracture models with a normal healing profile (74). At this stage, cartilage is reabsorbed and the area between the newly formed woven bone is deposited with new bone marrow comprised primarily of hematopoietic stem cells. During this final remodeling phase, T and B cells reappear in the entire callus area with OPG producing plasma cells being the major infiltrating lymphocytes (74) (Figure 4A). At the site of bone modeling and remodeling, increased number of osteomacs are present at 4 weeks post fracture (wpf) and they form a distinctive canopy structure over osteoblasts and promote mineralization (102). Mast cells are present through 5–6 weeks post fracture and have a prominent role in bone remodeling. They are mostly found lining the convex side of the fracture, in close proximity to the osteoclasts and in the bone resorption sites. This suggests the influence of mast cells over osteoclasts and its contribution to callus resorption (64, 65). In murine models, the remodeling process is vastly accomplished between 28 and 63 dpf, and the bone regains its original architectural form. Although, the remodeling time can vary in other mammalian species as well as gender, age, site of injury and the fracture defect size are important factors to consider.

## Delayed Fracture Healing

While long-bone fractures in humans typically heal in about 17 weeks (standard healing) to 35 weeks (delayed healing) depending on the severity, ~16% of all fractures, in human patients subjected to surrounding soft tissue damage and/or high energy trauma, results in non-union i.e., a failure to heal within 35 weeks of fracture (104). Moreover, patients with severe fractures accompanied by post-injury associated dysregulated inflammation or a pre-existing condition of chronic inflammation are at higher risk of complicated healing outcomes (Figure 4B). Dysregulated inflammation is triggered mainly by the trauma injuries and is not to be confused with the effects of pre-existing chronic inflammatory conditions that also cause impaired bone healing outcomes. Chronic inflammatory responses are mainly caused due to pre-existing conditions like muscular dystrophy (105), malnutrition (106), individuals with renal diseases (107), diabetes (108), smoking (109) and aging (110). Delayed fracture healing was also observed in HIV patients with a pre-existing immunocompromised condition (111). Clinical conditions following traumatic injuries that display delayed fracture healing and dysregulated inflammatory response include (i) isolated severe fractures, (ii) polytrauma and (iii) concomitant muscle trauma.

### Isolated Severe Fractures

Several studies have attempted to characterize the immune cell infiltration into the fracture site and their roles in animal models of delayed fracture healing. Functionally active macrophages or Osteomacs during the inflammatory phase are integral to bone union (88). Depleted Osteomacs during the initial stage disrupts the proper maintenance of hematopoiesis and osteoblastic function, thereby increasing the chances of impairing bone regeneration (102, 112). Another study was conducted to explain the upregulation of macrophage inhibitory factor (MIF) and its role in pathophysiology during fracture, demonstrated a delay in the mineralization of osteoid within the fracture callus and tremendously impaired fracture healing (113). Although the initial burst of innate immune cells determines the later stages of healing outcomes, it is important to note that the adaptive immune cells are also important regulators of endogenous bone regeneration. In a sheep model of delayed healing, there was a prolonged pro-inflammatory condition with increased number of cytotoxic T cells in the fracture hematoma and adjacent marrow as well as low expression of hematopoietic stem cell markers, heme oxygenase and VEGF (angiogenic factors) in the periosteum during the inflammatory phase, compared to normal healing, thereby causing delayed bone healing (114). A clinical investigation compared individual adaptive immune cell activation between patients (non-infected) with severe closed tibia head fractures demonstrating normal or delayed healing. The authors have reported increased number of a subset of CD8^+^T cells, i.e., CD8^+^T_EMRA_ cells (terminally differentiated effector memory) in circulation and in the fracture hematoma from patients with delayed healing, ranging from 1 to 18 weeks post-fracture, compared to those with normal healing, indicating a negative impact of these cells on healing outcomes [Figure 4B(i)]. The activated CD8^+^ T_EMRA_ cells with CD57^+^/CD28^−^ were predominantly seen in the fracture hematoma than in blood. In sterile conditions, the CD8^+^ T_EMRA_ cells respond to stress signals released from endothelial cells at the injury site or by pro-inflammatory cytokines, and not via the classical professional antigen presentation. Modulation of various effector function of CD8^+^T_EMRA_ cells greatly improved fracture healing, confirming the inhibitory effect of these callus infiltrating cells in bone repair. Importantly, the authors observed that the mice housed in specific pathogen free facilities had less CD8^+^T_EMRA_ cell counts with little to no role in delayed fracture healing, indicating that these memory cells appear in higher counts in individuals with pre-clinical conditions. Thereby, the authors concluded that the increased population of CD8^+^T_EMRA_ cells in delayed healing reflected the patient's individual immune response and not influenced by the fracture or surgery procedures (115). Despite the lack of direct connection of CD8^+^T_EMRA_ cells with the fracture, their presence in fracture patients could be used as an indicator to predict delayed fracture healing and could also represent a potential therapeutic target to achieve timely bone healing. Further investigation about the clarity of this response and its benefits in healing would be helpful. Importantly, the complexity and magnitude of injury determines the fate of innate and adaptive cells, in terms of activation, polarization and differentiation.

### Polytrauma

Polytraumatic patients, with an injury severity score of >15, have a combination of blunt chest trauma, open or closed fractures, infection, hemorrhage and/or burns. The incidence of combined trauma is accompanied by dysregulated systemic and local inflammatory responses that does not achieve resolution and has been identified as one of the multi-factorial causes of impaired/delayed fracture healing (2, 116). Delayed fracture healing or non-unions is significantly higher in patients with polytraumatic injuries compared to those with isolated limb injuries (58, 104). In most cases, the polytrauma-fractured bones have reduced callus volume and decreased bending stiffness. Approximately 40% of the polytrauma patients who suffer from delayed healing or non-unions demonstrate significant alterations of circulating leukocytes for at least 2 weeks post-injury (2, 104). In contrast to patients with isolated fractures, the patients with multi-trauma had less number of circulating neutrophils at 2–5 dpf and 11–14 dpf and increased number of local neutrophils during those times were reported within the fracture hematoma (2, 58). Similar temporal patterns of circulating monocytes were reported in multi-trauma patients i.e., 3 and 10–14 dpf (2) [Figure 4B(ii)].

While, burn polytrauma patients are often present with fracture injuries, there is little guidance available for the management of orthopedic trauma in combination with burn injury and needs further investigation (6, 117, 118). Blunt chest trauma (BCT) or thoracic trauma is one of the most prevalent injuries faced by polytrauma patients, which disturbs the systemic and local inflammatory balance and clinically is shown to delay fracture healing in humans and has been confirmed in laboratory mouse and rat polytrauma models with BCT and an osteotomy (119–121). Immediately following a BCT there is an overwhelming increase in the number of pro-inflammatory neutrophils and alveolar macrophages in the lung and this aggravated response negatively impacts the host's physiological health status and causes poor fracture healing outcomes (2, 122). In a rat model of polytrauma, the area of the newly formed bone was decreased in the BCT group at 35 dpf, suggesting that the initial healing process was normal, but gradually decreased after the soft callus formation phase (i.e., 5–16 dpf) (121). In the same polytrauma model, Recknagel et al. demonstrated that the impact of BCT significantly decreased macrophages at 3–7 dpf [Figure 4B(i)] and rapidly increased polymorphonuclear leukocytes (PMN) infiltration and IL6 expression within the early fracture periosteal callus, thereby postulating that this dysregulation of early inflammatory cells could have a negative role during later healing phases, the reason for delayed fracture healing (120). The neutrophil granulocytes readily respond to traumatic injuries and are the most abundant cells seen in the fracture hematoma within 24 h following multi-trauma. Neutrophils actively clear DAMPs and recruit monocytes to the injury site through secretion of chemo-attractants like MCP-1 (119). However, these neutrophil granulocytes are not the primary inducers of delayed fracture healing. Though neutrophils are recruited in significant numbers following trauma, neutrophil depletion studies in a rodent model of multi-trauma have demonstrated that, while neutrophil depletion mitigated pulmonary pro-inflammation, this resulted in elevated numbers of F4/80+ cells/monocytes invasion in the fracture hematoma, and ultimately offered little to no improvement in bone regeneration (119). The authors of this study concluded that while their presence does not prove deleterious to healing, the depletion of neutrophils from may disrupt the healing process, result in less favorable fracture healing outcomes caused by excess invasion of monocytes and pro-inflammation (123). While the impact of neutrophils on fracture healing appears minimal, excessive infiltration and activation of these cells following traumatic injury can result in damage to the bystander and remote organs via the release of excessive DNA, reactive oxygen species and matrix metalloproteinase (124, 125). This brings to light the importance of regulating the neutrophils to maintain their optimal numbers via novel therapeutic interventions. Detailed information about the underlying mechanisms and the clinical implications of neutrophils within the fracture hematoma during the fracture healing process after severe trauma is limited and needs further investigation. Additionally, systemic alteration of lymphocyte kinetics in polytrauma subjects is also associated with impaired bone healing outcomes (2, 120). In another study, Mangum et al. reported that polytrauma rat models demonstrated significant decreased bone volume fraction, inability to regain pre-surgery weight, altered inflammatory and osteogenic pathway gene expressions as well as heightened early systemic monocytes and granulocytes; and suppressed circulating lymphocytes at 1–3 dpf compared to single osteotomy (3) [Figure 4B(ii)]. Further, Amara et al. demonstrated the altered expression of C5a receptor and complement regulatory proteins on circulating neutrophils and monocytes, but not lymphocytes, post-polytrauma up to 10 dpf (126). Further illustrating the importance of the complement system in polytrauma. Recknagel et al. demonstrated that a C5aR antagonist significantly reduces the deleterious effect of a BCT on fracture healing outcomes (127). Contrastingly, the expression of C5aR decreases in single trauma, suggesting that aberrant increases in complement activation contributes to pathogenesis of delayed union. Taken together, polytrauma drives perturbed inflammatory response and results in drastically impaired healing time with the endpoints resulting in delayed healing and non-unions.

### Concomitant Muscle Loss

Certain polytraumatic injuries involve a fracture and significant volumetric muscle loss (VML), i.e., irrecoverable muscle injury occurring adjacent to the fracture site. VML is a debilitating condition and the associated hemorrhage that occurs with VML can be life threatening. Although ~250,000 civilians suffer per year from open fractures involving a component of VML injury, the occurrence of VML injuries is most prevalent and a major concern in wounded military soldiers accounting for 65% disability in soldiers in the orthopedic cohort (128). Importantly, the occurrence of VML leads to complete loss of contractile tissue and diminished mechanical strength within the muscle, and when this injury occurs within the setting of fracture, it leads to decreased bone mineralization in the fracture callus and delays bone regeneration. Furthermore, the development of extensive fibrosis at the VML site leads to instability of the affected fractured limb, while the loss of blood circulation and prolonged influx of immune cells contributes to delayed unions, having deposition of cartilage within the fracture callus instead of woven bone. The loss of bone regeneration in concomitant VML injury emphasizes on the importance of intact musculature adjacent to and surrounding a fracture for its timely healing. While instability of the injury and loss of vascularization contribute to impaired fracture healing, recently, studies have implicated an inflammatory response that is unbalanced and temporally disrupted in the VML muscle can delay fracture healing (41). Typically the inflammatory response in VML injury is triggered by a heightened release of endogenous DAMPs from damaged muscle fibers that activate toll like receptors and the inflammasome, thereby initiating cell signaling pathways to drive pro- and anti-inflammatory responses (129). Hurtgen et al. extensively characterized the immune response in the VML muscle and facture callus and assessed whether concomitant fracture/osteotomy altered macrophages and T cells infiltration in the injured muscle adjacent to osteotomy defect or vice versa (41). Following osteotomy and VML injury, the CD3^+^ T cells in injured muscle revealed the influx of CD4^+^ T cells and CD8^+^ T cells (predominantly CD4^+^ T cells) into the muscle injury site at 3 dpf through 28 dpf, with a peak at 14 dpf, compared to isolated osteotomy (41) [Figure 4B(iii)]. Further investigation on macrophages in the VML muscle from concomitant osteotomy group had extensive macrophage infiltration with mixed M1/M2 phenotype (predominantly M1) from 3 to 28 dpf compared to isolated osteotomy (41). Although the expression levels of recruited M1 and M2 in the muscle were mixed up to the initial 28 dpf, the number of macrophages with M1 phenotype was elevated and prolonged from 14 to 28 dpf, indicating that VML injury results in a largely M1 immune response in the concomitant osteotomy group (41) [Figure 4B(iii)]. This response was correlated with a significant increase in expression of genes associated with acute phase response, increased activation of the inflammasome, heightened cytokine/chemokine production, and enhanced receptor expression for inflammatory cytokines (41). M1 macrophages, produce reactive oxygen species (ROS) and pro-inflammatory cytokines like IL1β, IL6, and TNFα, and support pro-inflammatory T helper 1 (Th1) cells. Whereas, M2 macrophages produce anti-inflammatory cytokines like IL4, IL10, IL13, IL1R antagonist decoy IL1 receptor type II, growth factors like VEGF, TGFβ, and IGF1, and support anti-inflammatory T helper 2 (Th2) cells. Together, these interactions create a bridge between the innate and adaptive immunity during severe bone and muscle injuries. The heightened inflammation within an injured muscle can directly influence the immune infiltration within the adjacent fracture defect. In the fracture callus, from the concomitant VML group, the CD68^+^ monocyte/macrophages and CD3^+^ T cells were low at 3 and 14 dpf, respectively; and significantly high at 14 and 3 dpf, respectively, compared to single osteotomy group (41). Additionally, in sterile trauma induced systemic inflammatory response, the recruitment of adaptive immune cells to the site of injury is of great interest. Hurtgen et al. reported the upregulation of genes expressing co-stimulatory molecules required for T cell activation via the T cells and the antigen presenting cells (APCs) interactions in injured muscle from a composite injury rat model compared to uninjured muscle from osteotomy rats (41). Typically, in non-sterile/infected wounds, the APCs interact with T cells to activate and recruit them to the site of injury. However, in a sterile trauma condition the APCs digest endogenous molecules or alarmins such as TNFα, defensins, high mobility group box-1 protein (HMGB-1) and heat shock protein into small peptides and present them to T cells at the injury site, thereby creating a cross link between the innate and adaptive immune systems (130, 131).

Some of the other pre-existing chronic inflammatory response mounting factors that influence delayed union include: (i) Diabetes; (ii) Geriatrics and (iii) Smoking.

### Diabetics

Diabetes mellitus (DM), in particular type 2 DM (T2D), is well documented to have a negative impact on fracture healing and diagnosis of T2D associated with increased risk of fracture injury, despite patients often having normal to high bone mineral density (5). Theoretically, T2D interferes with the structural integrity of bone, rendering it more brittle. The high glucose levels observed in diabetic patients lead to accumulation of advanced glycation end products (AGEs), which may interfere with normal collagen deposition, leading to abnormal mineral deposition and increased bone fragility (132). T2D is also associated with defects to cortical and trabecular bone, with diabetic patients exhibiting increased cortical porosity, leading to a compromised biomechanical state with increased risk of fracture (133). While AGEs are known to contribute to the development of disorganized collagen matrices, AGEs also bind to their receptors (RAGE) to induce oxidative stress and activate inflammatory cells (123). Further, AGEs accumulates in cortical bone, where they activate local immune cells, contributing to a pro-inflammatory microenvironment. This local inflammation, in combination with increased concentration of AGEs within the bone may contribute to the activation of osteoclasts while reducing osteoblast function (133). While AGE-RAGE signaling may interfere with bone mineralization, and contribute to both fragility and delayed healing, other inflammatory molecules can also cause delayed union in T2D patients. Interestingly, in TD2 wound macrophages are forced to remain in their proinflammatory (M1) phase due to a dysfunction in the phagocytic functions known as efferocytosis (134). A recent study of immunologically restricted patients, which included diabetics, found higher concentrations TNFα, IL6, IL1β, and IFNγ in both the fracture hematoma and surrounding bone marrow when compared to non-immunologically restricted patients (135). Furthermore, when compared to the cellular composition of a normal hematoma, the fracture hematoma of immunologically restricted patients was characterized by increases in monocytes, macrophages (M1), and hematopoietic cells with significant decreases in Tregs (135). TNFα is an inflammatory cytokine that is overproduced in multiple disease states, including T2D, and has been demonstrated to reduce diabetic wound healing (136). In an animal model of diabetes, TNFα inhibited fracture healing of long bones by inducing apoptosis and inhibiting the proliferation of MSCs, thereby inhibiting their regenerative potential (137).

### Smoking

The negative effect of smoking on fracture healing has been well documented (4, 16, 138–141). In addition to exhibiting poor union post fracture, smokers also have a reduced capacity for soft tissue healing and a higher risk of infection (142, 143). According to Bender et al., the risk of delayed non-unions was significantly higher in current and previous smokers group compared to nonsmokers (138, 144). Despite the growing evidence that smoking delays timely bone regeneration post-surgery or trauma, clarity about the mechanisms of cigarret smoke on bone healing is lacking. Besides direct impairment of tissue oxygenation and decrease of serum concentrations of growth factors, smoking has damaging effects on bone stability by reducing bone mineralization, collagen synthesis and general healing of tibia fractures and open fractures. Smokers have been shown to have lower bone density compared to non-smokers, while nicotine itself inhibits alkaline phosphatase and collagen production (145) causing an impact on cancellous bone (146, 147). Experimental delivery of nicotine through drinking water in a rabbit model, resulted in a decrease in bone stability and radiological callus formation (148). In the event of fracture, a pronounced effect of nicotine and the other 3,500 chemical substances found in the cigarette smoke are responsible for the comorbidities seen in smokers (149), along with tar, nitric oxides and carbon monoxide being among the most potent inhibitors. Furthermore, nicotine prevents proliferation of fibroblast and osteoblasts during fracture healing while also inhibiting the proper maturation of macrophages (150). The effect of smoking on the survival of dental implants is a major field of study especially because the bone healing around the implant is a complicated process (151). Angiogenesis is integral for bone regeneration and is directly affected by nicotine and thereby impeding revascularization of bone grafts (152, 153). Further, nicotine slows down blood flow and thereby stimulates the release of catecholamines from the central nervous system and activates vasoconstriction (154, 155). Carbon monoxide recognizes and binds to hemoglobin and displaces oxygen molecules, thereby contributing to reduced tissue oxygenation in peripheral tissues. In order to restore normal fracture healing, it is essential to maintain enriched nutrient and oxygen levels for enough blood supply and tissue oxygenation at the injury site. Nicotine reduces circulating levels of VEGF and has detrimental impact on micro-vascularization (156–158). Moreover, nicotine negatively effects certain intracellular signaling pathways like the JaK2–STAT3 and NFκB signaling pathways and inhibits the production of TNFα, thereby interfering with the anti-inflammatory cascade (159, 160).

### Geriatrics

Aging is associated with increased risk of fracture due to changes to bone mass and density. The innate and adaptive immune response is altered with age, often skewed toward a chronic pro-inflammatory state that has been referred to “inflammaging” (161). The chronic, low-grade inflammation seen in inflammaging is characterized by increased circulating cytokines such as TNFα, IL6, and C-reactive protein(CRP); a condition that has been reported even in healthy elderly adults (162). It should be stated that aging is associated with increased prevalence of age-related diseases such as hypertension, diabetes, and heart disease (163), all of which are known to contribute to low-grade systemic inflammation (164). However, even adjustment for comorbidities does not completely account for the increase in circulating cytokines in elderly patients (164), and higher circulating concentrations of TNFα, IL6 and CRP are associated with increased bone loss in an older cohort (162). In the setting of chronic-low grade inflammation seen in aging, it is likely that TNFα contributes to increased osteoclastogenesis, and possibly contributes to bone destruction (165). While increased systemic inflammation certainly contributes to bone fragility, changes to the cells of the immune system itself may contribute to altered or delayed bone healing. Immune cells like neutrophils, monocytes and macrophages are essential for fracture healing under normal conditions (166), however, aging may inhibit their ability to initiate the appropriate inflammation cascade and subsequent resolution phase. For instance, compromised neutrophil chemotaxis in older patients could potentially increase damage to non-target surrounding tissues due to release of elastases to facilitate chemotaxis. Following fracture, a delay in neutrophil infiltration may also prolong inflammation and delaying the onset of resolution (167). Furthermore, monocytes numbers increase with age, and in aged mouse models they have been shown to exhibit enhanced inflammatory response (167). In a separate study, pharmacological inhibition of macrophage activation in aged mice resulted in increased callus volume following fracture (168). Paradoxically, recent data indicate that while the elderly population exhibits increased incidence of fracture, these patients may also be at lower risk for non-union. The authors of a recent study involving ~5,000 non-unions found that incidence of non-union increased with age until ~35–44 years of age, after which there was a steady decline in incidence of non-union (169). The authors do not speculate on the potential reason for the decline in incidence of non-unions in the elderly, but it may be partly due to a decrease in risk-taking behavior and less traumatic injuries in the older population.

## Therapeutic Interventions for Bone Healing

The knowledge of the impact of diseased states on healing is important to target specific treatments to correct abnormalities in patients. Novel therapeutic interventions have the potential to reduce morbidity, complications and may represent more economically feasible treatment strategies. A properly regulated inflammatory response (in a balanced and undisrupted manner) is a key requirement for efficient and timely bone fracture healing. Temporal disruption of immune cells in the fracture callus can be detrimental to all the four stages of bone healing and modulating inflammatory response in sterile trauma-induced conditions through therapeutic interventions has been a challenge. Some of the past interventions extend from targeted inhibition of receptor molecules to enzymatic removal of naturally occurring endogenous molecules that trigger chronic inflammation (115, 170, 171). A number of immunomodulatory therapies are under investigation for their ability to improve fracture healing, while other medications have been identified as potentially detrimental to fracture healing. NSAIDS and glucocorticoids, for example, have been reported to have a neutral or negative impact on fracture healing, and usage of this class of drug may be contra-indicated during fracture healing (172). Furthermore, while the selective depletion of deleterious immune cell populations is an appealing target, this must be investigated with caution. Non-specific depletion of neutrophils and macrophages immediately after trauma has been demonstrated to reduce fracture healing, while delayed depletion of macrophage and monocyte populations at later time points not only fails to improve healing outcomes, this practice may render a patient susceptible to infection without any perceivable benefit (119, 166). Modulations of specific macrophage subsets, as opposed to global depletion, may be feasible to guide the healing cascade, and investigations into the adoptive transfer of CD4^+^ T_reg_ cells have provided promising results (173–175). Additionally, the blockage of cytokines and DAMPS, like IL-6 and HMGB1, or their signaling cascades, as well as the supplementation of specific anti-inflammatory cytokines, like IL-17 and resolvins, have all been reported to improve wound healing outcomes (170, 176, 177). Though many molecules and therapies are the subject of *in vivo* research, approved immunomodulatory therapies to improve fracture healing are lacking. We have presented a selection of recently described potential immunomodulatory therapies with reported positive impacts on bone and fracture healing in Table 1. These therapies have been categorized into the following treatment modalities: cytokine blockage, anti-inflammatory therapy, cellular depletion, and cellular injection therapies. While some non-immunomodulatory therapeutic interventions proved successful, the treatments that are available need further improvement. We have listed some of these therapeutics in Table 2. We have also discussed some of the therapeutic interventions for improving fracture healing outcomes.

**Table 1:** Immunomodulatory therapies for fracture healing.

**Category(ies)**	**Therapy**	**Species; mechanism of injury**	**Route and timing of administration**	**Mechanisms of action/proposed mechanism of action**	**Cellular and/or structural and mechanical effects**	**Influence on fracture union**	**References**
Cytokine blockage	Soluble glycoprotein 130 fusion protein (spg130Fc) OR Anti-IL-6 antibody	12-week-old male C57BL/6 J mice; Femoral osteotomy with thoracic trauma	IP injection; 30 min and 48 h post injury	(Proposed) Specific inhibition of IL-6 or inhibition of IL6 trans-signaling will improve fracture healing	Spg130Fc administration enhanced fracture gap bridging and improved bending stiffness of the fracture callus	Inhibition of IL-6 trans-signaling, but not global inhibition of IL6, improves fracture healing in a model of non-union caused by severe trauma	(170)
Cytokine Blockage/Anti-Inflammatory Therapy	Biomimetic Anti-inflammatory Nano Capsule (BANC) coated with cytokine receptors and loaded with Resolvin D1 (RvD1)	8-week-old female C57BL/6 mice; 1 mm femoral bone defect	Delivered at the time of injury in boron-containing mesoporous glass scaffolds	Capsules were coated with lipopolysaccharide-treated macrophage cell membranes expressing cytokine receptors to neutralize inflammatory cytokines. BANCs were later activated by near-infrared laser irradiation, causing release of RvD1 and promotion of M2 macrophage polarization	Enhanced osteogenesis, as determined by reduced collagen staining	Hisological staining indicated reduced CD11b infiltration immediately following injury, increase M2 polarization within the defect, and increased bone formation	(174)
Anti-Inflammatory Therapy	Resolvin E1	5- to 6–week-old C57BL/6 mice; Osteolysis model receiving calvarial TNF-α injections	Daily IP injections of 50 ng RvE1 for 7 days after injury	RvE1 mediated resolution of inflammation would reduce osteoclastogenesis and reduce bone resorption	RvE1 decreased RANKL levels in osteoblasts and reduced expression of genes under the regulation of IL-6	Fracture healing was not assessed, but bone resorption was reduced in this model	(177)
Anti-Inflammatory Therapy	Iloprost	12-week-old female C57BL/6N micee 0.7 mm femoral defect	Delivered at time of injury via fibrin scaffold	Downregulation of CD8^+^ cytotoxic cells as well as the decreasing CD8^+^ cytokine profile would will improve fracture healing	Enhanced mineralization of MSC derived osteogenic cells	Improved healing outcomes, as evidenced by increased bone volume, total callus volume, and increased BV/TV	(178)
Anti-inflammatory Therapy	Local administration of IL-4 and IL-13	12-week-old female C57BL/6N mice; 0.7 mm femoral osteotomy	50 ng IL-4 and IL-13 applied to a collagen scaffold inserted into the osteotomy gap at the time of injury	Local administration of IL-4 and IL-13 were hypothesized to enhance M2 macrophage phenotype	Isolated BM macrophages exhibited a strong M2 polarization response to IL4/IL-13 stimulus	μCT analysis indicated improved callus and bone volume compared to wild type	(166)
Cellular depletion (Adaptive)	Selective depletion of CD8^+^ T cells with anti-mouse CD8 antibody	12-week-old C57BL/6N mice; 2 mm femoral osteotomy	Delivered four consecutive days and immediately before the surgery	Depletion of CD8^+^cells will diminish the effect of memory CD8^+^T_EMRA_ cells response, which have a negative impact on bone healing	Memory CD8^+^T_EMRA_ cells are potent producers of IFNγ and TNFα, which inhibit osteogenic differentiation and survival of bone marrow MSCs	Depletion of CD8^+^ T_EFF_ cells results in improved fracture healing outcomes	(115)
Cellular injections	Platelet rich plasma	Prospective Randomized Study (*n* = 72, 69 males, 3 females); Acute femoral fractures receiving IM nailing; with or without PRP injection/application	Closed IM nailing with PRP injection at fracture site OR PRP gel and fibrin membrane applied to fracture site of open IM nailing	Platelet release growth factors, such as TGFβ1, along with the stability provided by a fibrin membrane may accelerate healing in an open fracture	PRP appeared to accelerate fracture healing, likely through a short-term increase in osteogenesis	PRP appeared to accelerate fracture healing, as evidenced by increased cortex to callus ratio at 3- and 4-months post injury, regardless of nailing technique. This increase in healing was no longer significant after 6 months	(171)
Cellular injections	Adoptive transfer of T regulatory cells	12-week-old female C57BL/6 mice kept under specific pathogen conditions; 0.7 mm non-critical femoral osteotomy	CD4+ regulatory T cells were isolated by magnetic activated cell sorting then injected into the tail vein prior to injury	An increased CD4^+^ T_Reg_ to CD8^+^ T_EFF_ ratio will improve fracture healing by reducing the negative impact of CD8^+^ cells	A significantly higher ratio of CD4^+^ T_Reg_ to CD8^+^ T_EFF_ was observed in animals following adoptive transfer	μCT analysis indicated significantly increased BV/TV in the femora of animals receiving osteotomy and adoptive transfer of CD4^+^ T_Reg_ cells	(175)

**Table 2:** Therapies for improving fracture healing outcomes.

**Therapy**	**Species; mechanism of injury**	**Route and timing of administration**	**Mechanism of action; proposed mechanism of action**	**Structural and mechanical effects**	**Influence on fracture union**	**References**
NELL/PEGylated NEL-like protein	10 weeks old CD-1 mice; Open osteotomy of bilateral radii	Weekly, systemic administration	Activation Wnt/B-catenin signaling pathway through integrin β1-receptor; Enhanced osteoblastogenesis; reduced osteoclast staining within the callus	Improved bone mineral density at the fracture site; Elevated remodeling activity at the fracture site; Enhanced angiogenesis and vascularization	Accelerated callus union compared to control (PBS)	(179)
Exosomes from Human Bone Marrow Derived Stem cells	C57BL/6 WT mice and CD9^−^/^−^ C57BL/6 mice; Transverse femoral shaft injury by three-point bending	Injection of isolated exosomes into the fracture site at 1 and 8 dpf	(Proposed) Enhanced endochondral ossification, enhanced stem cell homing to the fracture site, induction of osteogenesis and angiogenesis following exosomal miRNA delivery	Enhanced callus formation in CD9^−^/^−^ mice at 2 weeks; Bone union in CD9^−^/^−^ mice by 3 weeks; Enhanced vascularization in CD9^−^/^−^ mice; Accelerated bone union in WT type mice	Reduced delayed fracture healing in CD9^−^/^−^ mice	(180)
Bone targeting liposome formulation of Salvianic Acid A (SAA-BTL)	12 weeks old female CD1 mice; Prednisone induced delayed union in a closed femur fracture model	Administered locally 3 dpf then weekly for an additional 18 dpf	SAA increases angiogenesis and increases osteocyte lacunar canaliculi while reducing adipogenesis in bone marrow; Stimulates osteogenesis through regulation of RANKL, BMP, Wnt/β-catenin	SAA-BTL improved stiffness, ultimate and yield stress, and flexural modulus	Treatment with SAA-BTL significantly shortened fracture healing time by potentiating osteogenesis, angiogenesis and cartilage mineralization within the callus	(181)
Recombinant Vascular endothelial growth factor (rhVEGF)	12–15 months old beagles; Delivered via coralline-nanohydroxyapetite scaffold bone substitute	VEGF enhances vascularization; (Proposed) nHA/coral scaffolds would promote osteointegration	Mandibular defect	Nanohydroxyapetite-coralline blocks coated with rhVEFG promoted neovascularization	Failed to enhance bone formation	(182)
Fresh or Freeze-Dried Platelet Rich Plasma (FD-PRP)	8 weeks old Sprague-Dawley rats; Delivered via powdered artificial bone (hydroxyapatite-collagen composite)	Bilateral-posterolateral fusion	Bilateral-posterolateral fusion	FD-PRP treatment achieved similar trabecular formation and mechanical strength as BMP treated control	Accelerated bone union was observed in groups receiving FD-PRP	(183)

### Non-immunomodulatory Therapies

Bone is a complex tissue that requires many nutrients, especially during fracture repair, and multi-nutrient therapy has been suggested to improve fracture healing (184, 185). Dietary supplementation with vitamin C, bioflavonoids and flavonols (quercitin and proanthrocydins), and omega-3 fatty acids is associated with improved regulation of the inflammatory response and expedited healing (186–188). Likewise, several studies have demonstrated that multi-nutrient therapy (that includes protein, carbohydrates, amino acids, sodium, potassium, calcium, magnesium, chloride, trace minerals, and fat soluble vitamins) reduces complication and accelerate fracture healing, as well as reduce complications and mortality (189–191). Similarly, antioxidant therapy may reduce extensive oxidative damage to the bone and surrounding tissue caused by free radicals. These free radicals are associated with inflammation, further breakdown of bone collagen and excessive bone turnover. Antioxidants are known to repair such oxidative damage (192). However, increased free-radical production can curb the effects of the natural antioxidant defense mechanisms. In such cases, exogenous antioxidants including vitamins E and C, lycopene, and alpha-lipoic acid are beneficial in suppressing the destructive effect of oxidant free radicals systemically and improving fracture healing in animal models (192).

### Pro-resolving Lipid Mediators

As mentioned earlier, a well-balanced inflammatory response is an integral component in the bone healing process. Inflammatory mediators are responsible for maintaining and keeping this balance. Cells at the site of trauma release large amounts of endogenous inflammatory prostaglandins and eicosanoid derivatives called pro-resolvin/lipid mediators, generated through activation of cyclooxygenase (COX) enzymes, COX-1 and COX-2. Prostaglandin-induced inflammation is an essential component of the fracture healing process, and COX-1 and COX-2 play important roles in fracture healing. Endogenous lipid mediators in lieu with M1 macrophages are a natural and integral part of osteogenesis because they maintain a tight regulation of the initial inflammatory immune response which is crucial to timely fracture healing (193). In cases of severe trauma, exogenous administration of synthetic pro-resolving lipid mediators like resolvins (177) and maresins (194) could help promote early resolution of the injury rather than inhibit inflammatory response, which could be a promising future therapeutic to restore fracture healing.

### Scaffolds or Bio-Regenerative Materials

Nanoparticle modified composite scaffolds (195) and bio-regenerative materials (196) have recently attracted great interest as promising candidates for supporting bone regeneration. The use of scaffolds to promote bone healing have tremendously increased in the last two decades for drug and gene delivery systems into the body, to provide physical stability to the bone and a suitable environment for bone regeneration (197, 198). Essentially, scaffolds must be able to induce rapid cell adhesion, proliferation and differentiation while possessing proper mechanical strength to sustain physiological loads. Bio-engineered materials like biodegradable polymers and ceramics have been successful in mimicking the natural bone, although they have a few limitations in terms of mechanical strength (199). Those limitations have been resolved to a certain extent by incorporation of nanoscale organic and inorganic materials into biodegradable polymers scaffolds (200) as well as creating suitable surface modifications to promote a favorable environment for immune modulation and allowing for eventual bone regeneration [reviewed in Lee et al. (201)].

### Tacrolimus (FK506) Mediated Immunomodulation

FK506 is a FDA approved immunosuppressive compound with osteogenic effects (202–204) that can impair IL2 dependent T cell activation by inhibiting enzymatic activity of calcineurin and subsequent translocation of nuclear transcription factors required for T cell maintenance. Importantly, FK506 regulates early inflammation and rescues fracture healing in a rat model with concomitant muscle trauma/VML. Treatment with FK506 reduced macrophages and T cells infiltration within the injured muscle at 3 dpi. At 14 dpi macrophage infiltration was rescued, however, T cell levels remained suppressed within the injured muscle and the fracture callus. Most importantly, FK506 rescued fracture healing and recovered mechanical properties in rats with concomitant muscle trauma. While, in rats without concomitant muscle trauma it did not fully recover mechanical properties of the isolated fracture. These observations demonstrate the detrimental effect of both, the enhanced T cell infiltration in the muscle injury site in the absence of treatment and the FK506 mediated T cells suppression in isolated fractures, during the fracture healing process (21). Thus, suggesting the importance of maintaining optimal levels of T cells for normal fracture healing. Improved therapeutic interventions that regulate T cell recruitment and activation could be promising in orthopedics. While, FK506 has a beneficial role in sterile musculoskeletal trauma related fracture healing outcomes, the limitations due to its T cell suppressive role and lack of anti-bacterial/viral role includes opportunities for infections that could have further detrimental effects in trauma patients (205, 206). Moreover, apart from its T lymphocyte-suppressive role, FK506 has been shown to directly mediate osteogenic differentiation of mesenchymal stem cells *in vitro* in the presence of BMP (202) and may augment challenged fracture healing *in vivo* (207).

### Minced Muscle Grafts

Another recent advancement is the administration of minced grafts as potential therapeutics to restore VML, due to the ability of the minced grafts to promote fiber regeneration in wounded muscles (208). Moreover, in a rat model of musculoskeletal trauma, minced muscle graft mediated VML injury repair modulates the inflammatory response and has shown to improve endogenous fracture healing and muscle strength (209).

### Mesenchymal Stromal/Stem Cells (MSCs)

MSCs function by orchestrating the host's cellular and molecular response to injury. The use of exogenous MSCs in fracture unions is one of the most widely studied therapeutic approach, with on-going pre-clinical trials investigating their ability to improve non-union fracture healing (210). The source of MSCs is an important consideration in terms of targeted therapeutic interventions. Studies have reported that exogenous implantation of bone marrow derived MSCs into the fracture site acts as seeds/precursors to differentiate into bone healing cells (osteoblasts and chondroblasts) and quickly transition from inflammatory phase to soft callus phase to regulate bone fracture repair (211, 212). Additionally, MSCs accelerate tissue regeneration by switching off early inflammatory responses via their trophic mechanisms and restore fracture healing. Improved and accelerated fracture healing is a result of the timely recruitment and proliferation of osteoblasts differentiated from pre-existing or implanted MSCs and their prior cross-talk with pre-osteoclasts at the fracture site during soft and hard callus formation (213). In a model of polytrauma, implanted MSCs improved healing of outcomes following liver and bone tissue injury (214). It is evident that the application of MSCs is a promising therapeutic approach for the clinical management of long bone fracture healing and non-unions; however, its administration still requires careful coordination with the ongoing healing process.

The therapeutic potential of exogenous MSCs has revolutionized the field of fracture care and many labs are still refining their application in the field. Much about MSCs and their beneficial role in fracture healing has been previously extensively reviewed at length (215).

## Conclusion

Developing highly effective interventions requires an in-depth knowledge about the immune response during the healing phase in injured muscles and bones, keeping in mind the different healing cascades and immune cells involvement. Recent advances in understanding of the molecular basis of healing and inflammation in complex wounds have provided crucial guidance for improving care for critically injured patients. In summary, we discussed the acute inflammatory response in normal/standard healing process of single trauma i.e., isolated wounds. Next, we discussed delayed healing impacted by dysregulated inflammation in muscle wounds and long bone fractures especially following severe isolated fractures, polytrauma and/or concomitant muscle loss as well as in the case of other comorbidities like diabetes, smoking and aging. We also highlighted recent advances in therapeutic measures for improving bone fracture healing.

Normal wound and fracture healing processes require a well-balanced inflammatory response. A shift in the normal balance can be detrimental to the healing outcomes causing impaired/delayed healing. Immunomodulatory therapeutic interventions can prove to be beneficial, if applied in right proportions and in a timely manner during the healing process. There is an urgent requirement for developing targeted immunomodulatory therapeutic interventions especially for delayed fractures associated with polytrauma and concomitant muscle trauma. As we continue to extrapolate new information about the cellular and molecular players of osteo-immunogenic responses associated with delayed fracture healing, the better prepared we will be to develop new therapeutic strategies to treat these costly clinical problems. Moreover, the precise underlying cellular mechanisms that drive bone healing need further attention. Finally, the understanding of basic immune-biological functions of healing will allow wound care specialists greater insights about the importance of how their skills can improve musculoskeletal wound healing processes.

## Author Contributions

PM refined the overall concept, wrote the first draft, and revised the manuscript. LM wrote sections of the manuscript and contributed to the revision. JW developed the broad focus and concept of the manuscript, and contributed to the revision. All authors read and approved the submitted version.

## Conflict of Interest

The authors declare that the research was conducted in the absence of any commercial or financial relationships that could be construed as a potential conflict of interest.
